# Imatinib treatments have long-term impact on placentation and embryo survival

**DOI:** 10.1038/s41598-019-39134-0

**Published:** 2019-02-22

**Authors:** Wael Salem, Kailiang Li, Christopher Krapp, Sue Ann Ingles, Marisa S. Bartolomei, Karine Chung, Richard J. Paulson, Romana A. Nowak, Lynda K. McGinnis

**Affiliations:** 1 Department of Obstetrics and Gynecology, Division of Reproductive Endocrinology and Infertility, University of Southern California, Los Angeles, CA USA; 2 Department of Preventative Medicine, University of Southern California, Los Angeles, CA USA; 3 Department of Animal Sciences, University of Illinois, Urbana-Champaign, IL USA; 4 Department of Cell and Developmental Biology, University of Pennsylvania, Philadelphia, PA USA

## Abstract

Imatinib is an oral chemotherapeutic used primarily to treat chronic myeloid leukemia (CML) and gastrointestinal stromal tumors (GIST). The potential effects of cancer treatments on a patient’s future fertility  are a major concern affecting the quality of life for cancer survivors. The effects of imatinib on future fertility are unknown. It is teratogenic. Therefore, patients are advised to stop treatment before pregnancy. Unfortunately, CML and GIST have high rates of recurrence in the absence of the drug, therefore halting imatinib during pregnancy endangers the mother. Possible long-term (post-treatment) effects of imatinib on reproduction have not been studied. We have used a mouse model to examine the effects of imatinib on the placenta and implantation after long-term imatinib exposure. We found significant changes in epigenetic markers of key imprinted genes in the placenta. There was a significant decrease in the labyrinth zone and vasculature of the placenta, which could impact fetal growth later in pregnancy. These effects on placental growth occurred even when imatinib was stopped prior to pregnancy. These results indicate potential long-term effects of imatinib on pregnancy and implantation. A prolonged wash-out period prior to pregnancy or extra monitoring for possible placental insufficiency may be advisable.

## Introduction

With the advent and use of tyrosine kinase inhibitors (TKI) over the course of the last decade, significant progress has been made in the treatment of multiple cancers^1–3^. Whereas conventional cytotoxic chemotherapeutic agents target rapidly dividing tumor cells, TKIs are more selective in targeting aberrant tyrosine kinases that are activated in certain cancers, including signaling pathways which impact the growth, angiogenesis, invasion and metastasis of cancers. Unfortunately, TKIs have also been associated with a variety of side effects including disruption of the thyroid, adrenal function, bone remodeling and gonadal dysfunction. It follows that collateral and unintended consequences may take place with the use of a TKI. The human genome is known to harbor more than 500 protein kinases. While TKIs are designed to be selective for single kinases of interest, the highly conserved ATP binding site within kinases enables “selective” TKIs to bind more than one kinase. These off-target interactions are of lower affinity than with the primary target. However, TKIs can also inhibit unintended tyrosine kinases and their downstream signaling pathways.

Imatinib Mesylate (Gleevec) is a tyrosine kinase inhibitor with specific activity against the fusion protein BCR-ABL however, it is also an effective inhibitor of ABL1, ABL2, KIT and PDGFR. It has been approved by the Federal Drug Administration (FDA) for chronic myelogenous leukemia (CML) and gastrointestinal tumors (GIST)^4,5^ and is being tested as treatment for numerous other cancers, including pediatric tumors^6^. Reproductive-aged individuals are often treated with imatinib but the potential effect on their future fertility and on subsequent pregnancy potential is unknown. At the molecular level, imatinib inhibits specific signaling pathways such as KIT and PDGFR, which are known to play a role in placental and gonadal development^7–12^.

Animal models and clinical data demonstrate the potential for teratogenic effects during pregnancy^13,14^. It is therefore the general recommendation to discontinue administration in pregnancy. Unfortunately, some women who have stopped imatinib prior to pregnancy have had a recurrence of their cancer in pregnancy leading to a very difficult evaluation of the risks and benefits of discontinuation^15,16^. Animal studies evaluating the potential teratogenicity of imatinib demonstrated multiple deformities including encephalocele, exencephaly and bony skull deformities in rats at doses of 45 mg/kg on a daily basis^17^. This dose is the equivalent of 400 mg/day, a dose well within the prescribed clinical dose window. Furthermore, the animal models (rats) in these studies had higher fetal resorption rates, spontaneous losses, nonviable pups and increased pup mortality. At doses more than 100 mg/kg there was a total fetal loss. Unfortunately, studies have not reported information on pregnancy or fetal development after imatinib cessation.

Because of the potential for birth defects and pregnancy losses, it is recommended that patients stop their imatinib treatments before attempting to become pregnant. However, high (>60%) rates of cancer progression are predicted for these patients during pregnancy^18^. Due to this contraindication between the teratogenicity of imatinib and cancer progression without imatinib, patients are advised to stop imatinib prior to pregnancy but is nothing known on how long a wash-out period is needed or safe^18^.

Pye *et al*. reviewed 180 women who became pregnant while taking imatinib^13^. First trimester exposure was found in 71% of the women while 26% had been exposed throughout their pregnancy. Outcome data was limited to 125 pregnancies. Of these gestations, 15% resulted in a spontaneous abortion while 28% ended in elective terminations. In sum, 9.6% (12/125) of the infants had fetal abnormalities including hydrocephalus, craniosynostosis, hypoplastic lungs, renal agenesis, exomphalos and scoliosis^13^. While the rates of certain fetal abnormalities were demonstrated to be much higher than would be anticipated in the normal population, it should also be stressed that the retrospective nature of this study makes it difficult to delineate long term sequelae of the children presumed to be normal at birth. The mechanisms by which imatinib caused fetal losses and abnormal development are unknown. Possible effects of imatinib at the fetal/maternal interface and placenta have not been studied. Concern also arises that epigenetic changes may be concealed when a previously presumed normal baby is born with the potential for long term health consequences.

There is a mounting body of literature implicating the role of epigenetic disturbances affecting poor fetal growth and adult disease^19^. The placenta is particularly sensitive to chemical and environmental exposures during pregnancy. These effects include changes in DNA methylation of critical imprinted genes, epigenetic changes in the placenta that may have long term implications for fetal health^20,21^. We hypothesized that imatinib exposure in utero may affect placental development and that halting imatinib prior to pregnancy would prevent these detrimental effects. To test this hypothesis we have used a combination of gene expression and histological analysis in a mouse model, in which mice were treated long-term with imatinib during the pregestational period.

## Results

### Experimental design

This study included 2 imatinib treatment regimens and one control (Table 1). One set of mice (4wk) were injected ip once each day with 400 mg/kg imatinib, plus they received imatinib ad lib in their drinking water (1 mg/ml) for 4 weeks (4wk) (see Supplementary Fig. S1 for analysis of imatinib concentration in mouse serum). At the end of 4wk of treatment, the imatinib was stopped, females were mated, then housed individually to E13 of pregnancy. The second group of females were treated with imatinib the same as the 4wk group, but this second group (4wk + p) continued to received imatinib in drinking water during pregnancy. The third group (controls) were injected daily ip with sterile water for 4 weeks prior to pregnancy.

**Table 1:** Treatment Groups.

Group 1: 4wk	400 mg/kg imatinib injected ip daily x 4 weeks + 1 mg/ml in drinking water BEFORE pregnancy only; NO imatinib during pregnancy
Group 2: 4wk + p	400 mg/kg imatinib injected ip daily x 4 weeks + 1 mg/ml in drinking water Continued imatinib in drinking water during pregnancy*
Control	Sterile water ip daily x 4 weeks

*In the 4wk + p group, imatinib treatment in drinking water was continued during pregnancy. IP injections were discontinued during pregnancy to avoid accidental injection into the expanding pregnant uteri and/or fetuses.

### Pregnancy analysis

The mean litter size was statistically different between the groups (Controls: 13.5, Group 4wk: 11.6, Group 4wk + p: 8.8 mean litter size, p = 0.048; Table 2). While the control group had no fetal resorptions observed, both treatment groups experienced resorptions detectable at E13 (mean resorptions per litter, Control: 0, Group 4wk: 1.67, Group 4wk + p: 0.27; p = 0.016). At the completion of the study, the mean weights of the mothers and their pups were not different between groups (mothers: Control: 40.8 g, Group 4wk: 38.7 g, Group 4wk + p: 39.7 g, p = 0.7; pups: Control: 76.9 mg, Group 4wk: 65.5 mg, Group 4wk + p: 63.1 mg, p = 0.3). In summary, there was a decreased litter size and increased resorption rate in the imatinib treated mice. Mice treated with imatinib before and during pregnancy had the lowest number of pups but fewer visible resorptions. Therefore, embryos in this group were lost very early in pregnancy, possibly prior to implantation or there may have been a lower number of oocytes ovulated. Even when imatinib was stopped prior to pregnancy (4wk), there was a significant effect of the long-term imatinib exposure on subsequent pregnancies.

**Table 2:** Pregnancy data of control and study mice.

	Control n = 8	4wk n = 9	4wk + p n = 12	P=
Females pregnant	100% (±0)	89% (±27)	75% (±28)	NS
Mean Litter Size/female	13.5 (±5.7)	11.6 (±5.4)	8.8 (±7.6)	0.048
Resorptions	0 (±0)	1.67 (±1.3)	0.27 (±1.6)	0.016
Mean female weight (g)	40.8 (±4.6)	38.7 (±4.5)	39.7 (±3.8)	0.70
Mean pup weight (mg)	65.5 (±14.0)	63.1 (±13.4)	76.9 (±26.8)	0.30

n = number of females; one-way ANOVA; all values reported as means ± standard deviation (SD).

### Hormone levels

**Figure 1:**
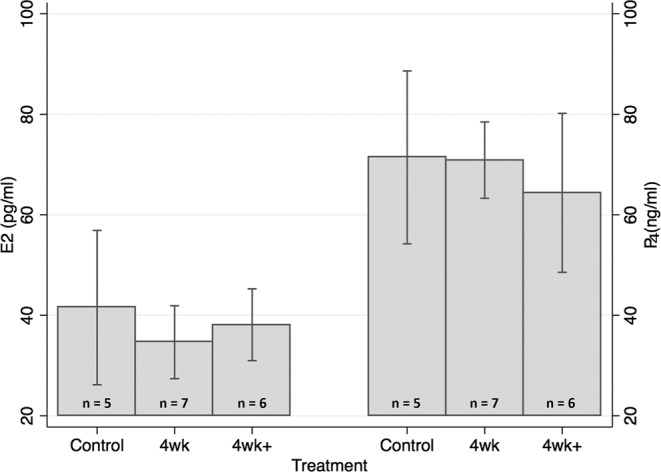
Maternal Estradiol and Progesterone. Serum was collected from females at the time of euthanasia. Estradiol and progesterone were measured by immunoassays on an Immulite 2000. E2 = estradiol (pg/ml), P4 = progesterone (ng/ml), n = number of serum samples assayed, one-way ANOVA compared treated groups versus control; Error bars = SD.

Estrogen and progesterone were measured in maternal serum at E13 but no significant differences were detected (Fig. 1).

### DNA methylation analysis

To investigate the possibility of gene expression effects of imatinib on placentation, we first examined DNA methylation of specific placental genes by bisulfite pyrosequencing. A total of three placentas from each of three mice within each treatment group was used for the analysis (n = 9/group). We targeted seven differentially methylated imprinting control regions (ICR) including *H19* differentially methylated region (*DMR*), *Ig DMR*, *Peg1 DMR*, *Peg3 DMR*, *Kv DMR*, *Snrpn DMR*, *and Igf2 DMR1*; genes important in placental function and with known sensitivities to environmental impacts^22^. The mean methylation of all CpG sites was reported for each ICR. Six of the genes under investigation demonstrated a statistically significant difference across the three treatment groups (Table 3).

**Table 3:** DNA methylation profile comparison.

	Control n = 9	4wk n = 9	4wk + p n = 9	P=
H19 DMR	52.05 (±0.65)	50.09 (±0.63)	52.83 (±0.74)	0.029
Ig DMR	40.60 (±0.94)	37.30 (±0.75)	42.07 (±0.80)	0.0022
Peg1 DMR	45.19 (±0.50)	41.67 (±0.78)	45.94 (±0.70)	0.0027
Peg3 DMR	50.36 (±0.60)	48.18 (±0.63)	50.93 (±0.63)	0.012
Snrpn DMR	44.16 (±0.71)	41.54 (±0.53)	44.71 (±0.60)	0.0017
Kv DMR	48.99 (±1.08)	48.80 (±0.27)	52.7 (±1.03)	0.0039
Igf2 DMR	44.49 (±1.96)	38.32 (±2.07)	41.73 (±1.90)	0.12
LUMA assay global methylation	52.39 (±1.47)	53.02 (±2.93)	47.53 (±1.91)	0.16

Means +/− standard error of the mean (SEM); n = number of placenta. Epigenetic data were evaluated using non parametric and was thus evaluated using a Kruskal-Wallis test.

Group 4wk placentas were significantly hypomethylated compared to controls at several allele specific ICRs (Table 4). *Snrpn DMR* (p = 0.009), *Peg3 DMR* (p = 0.02), *Peg1 DMR* (p = 0.003) and *IgDMR* (p = 0.009) were all hypomethylated in Group 4wk versus controls. The *Kv DMR* was hypermethylated in Group 4wk + p versus controls (p = 0.04). *Igf2* was not different between groups.

**Table 4:** Individual group comparisons of DNA methylation.

Comparison	H19 DMR	Ig DMR	Peg1 DMR	Peg3 DMR	Snrpn DMR	Kv DMR	Igf2 DMR
4wk vs control	0.058	0.0092	0.0031	0.024	0.0092	0.72	0.07
4wk + p vs control	0.40	0.20	0.40	0.43	0.31	0.046	0.57

p-values for direct comparison between imatinib treatments versus controls. Pairwise comparison between the study groups of interest and the control group was done using a Wilcoxon rank sum test.

### Global methylation

To ascertain global methylation within the placenta, a LUMA assay was used. LUMA exploits the MspI and HpaII restriction cut sites followed by polymerase extension and bisulfite pyrosequencing to measure global DNA methylation throughout the genome. Global placental methylation was approximately 50%, which is in line with previously reported data for these imprinted genes^23^. Moreover, there were no statistically significant differences between groups with respect to global DNA methylation (p = 0.17) (Table 3). This points to epigenetic errors confined to specific loci and not to an impairment of the global DNA methylation apparatus during placental development following imatinib treatments.

### Expression of imprinted genes

To determine if the changes in DNA methylation resulted in significant changes in RNA of the affected genes, we used Real Time qPCR. The *H19*, *Igf2*, *Peg3*, and *Kv* genes control the expression (RNA) of *H19*, *Igf2*, *Peg3*, *Cdkn1c*, and *Kcnq1ot1* and were thus investigated. *Kcnq1ot1* is a long non-coding RNA controlled by *KvDMR*. We detected no statistically significant differences in the levels of RNA between the genes examined (Supplementary Table S1). There was a large variation in gene expression between the samples within each group. A similar relationship between hypomethylated ICRs and apparently normal expression (RNA) of target genes has also been reported in other investigations^24^.

### Histological outcomes

**Figure 2:**
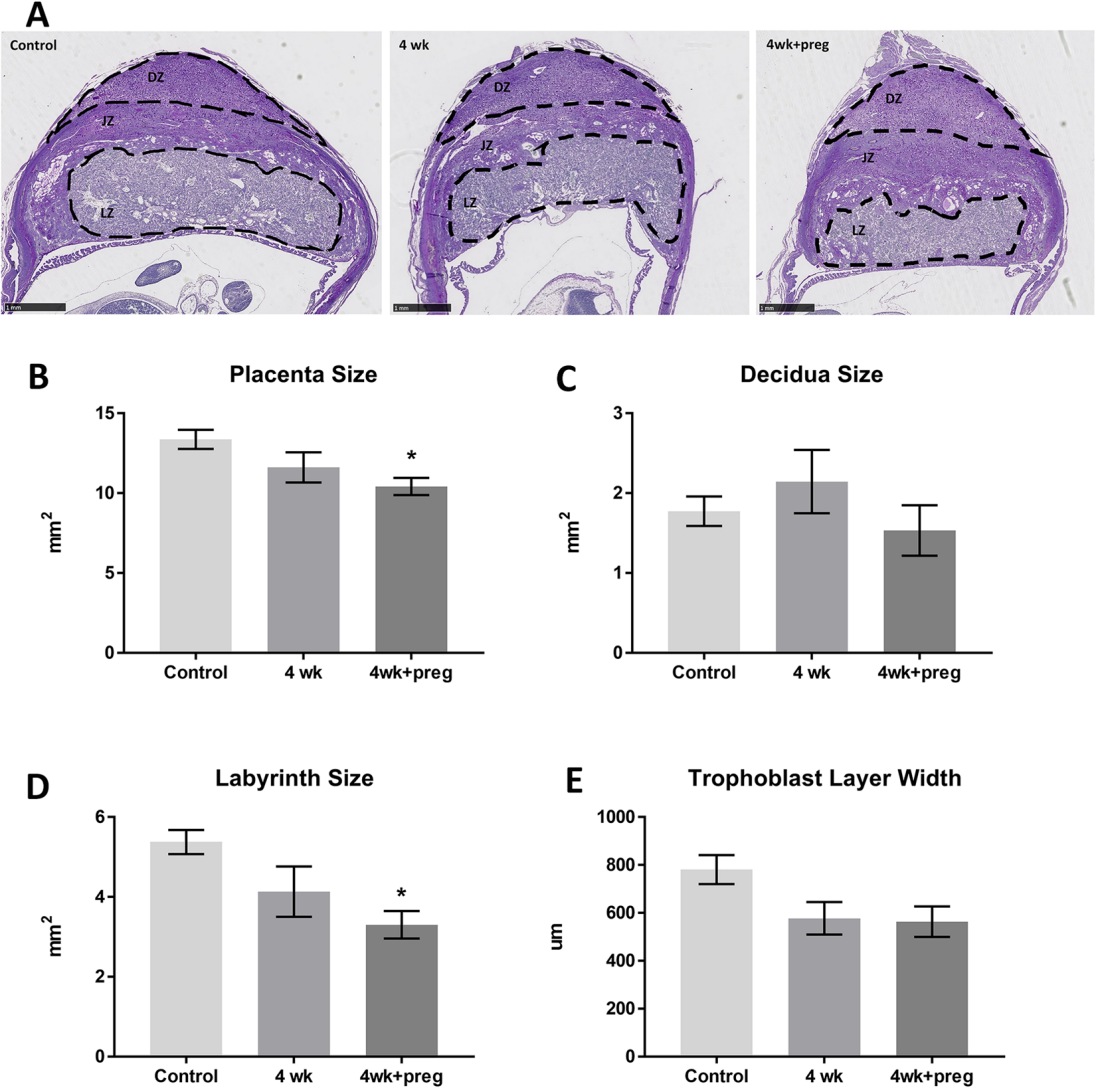
Imatinib treatment caused reduction in overall size of the placenta, especially within the labyrinth layer. (**A**) Tissue sections of placenta of each treatment group stained with H&E (n = 27 females; image bar = 1 mm) DZ: decidual zone, JZ: junctional zone, LZ: labyrinth zone. (**B**–**E**) Quantitative results of placenta size (**B**), decidual size (**C**), labyrinth size (**D**) and trophoblast layer width (**E**) of each group. *p < 0.03 compared to controls. n = 6–8 placentas per group, N = 4 animals per group. Error bars = SEM.

To further examine the possible effects of long-term imatinib on placentation, all three study groups were examined through careful histological analysis of the placentas (Fig. 2A). Females in group 4wk + p had significantly reduced size of the implantation site (placenta size; 10.4 vs 13.4 mm^2^ p = 0.025; Fig. 2B) and a smaller labyrinth zone (3.3 vs 5.4 mm^2^ p = 0.015) compared to controls (Fig. 2D). Group 4wk placenta and labyrinth size were numerically smaller than controls, but did not reach statistical significance (11.61 mm^2^ and 4.13 mm^2^, respectively; Fig. 2B,D). There were no differences in the overall size of the decidua (Fig. 2C) or spongiotrophoblast (data not shown) layers. The width of the trophoblast layer was numerically smaller for imatinib treated groups 4wk and 4wk + p, however these were not statistically significant.

**Figure 3:**
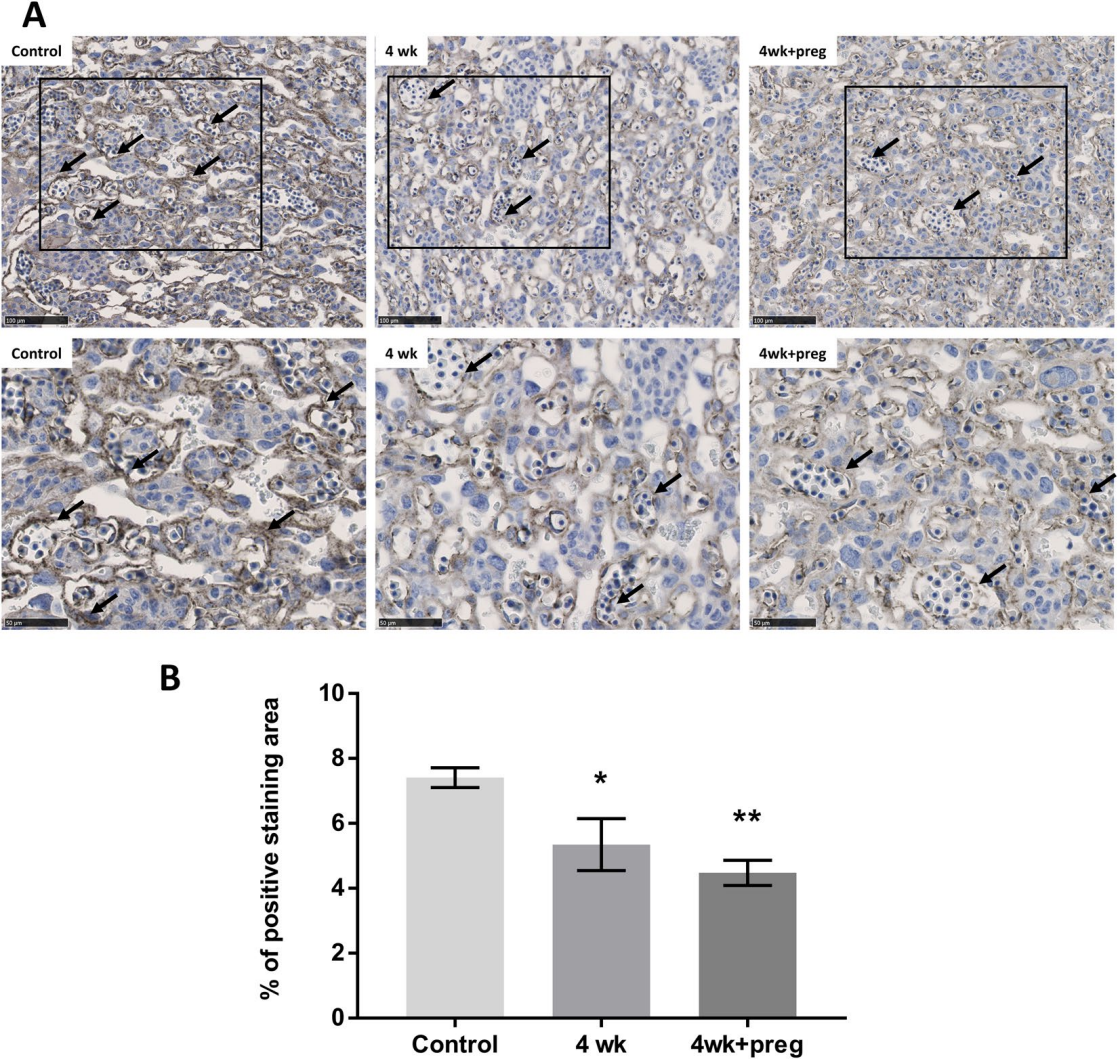
Imatinib treatments caused a reduction in angiogenesis within the placenta. Placenta tissue sections were labeled for anti-CD31 antibody to enable measurement of the vasculature within the labyrinth zone. (**A**) Representative images of the labyrinth zone of each treatment group. Top row = 20x to show overall area of tissue (bar = 100 µm); bottom row = 40x enlargements of the same images (bar = 50 µm), to visualize details within the tissue. Arrows indicate positive staining of CD31 + vessels. (**B**) Quantitative results of CD31 within each group. *p = 0.04, **p = 0.007. n = 4 females/treatment group, 6–8 placenta. Error bars = SEM.

The labyrinth layer of the placenta is comprised largely of endothelial cells and blood vessels. Therefore, to determine if imatinib treatments impacted angiogenesis within the labyrinth layer, tissue sections of placenta were labeled with antibodies against CD31 (PECAM-1), a marker of endothelial cells and vasculature. Representative images of the labeled tissue sections are presented in Fig. 3A (20x); the magnified (40x) shown below. Arrows indicate CD31 + vasculature. Imatinib treated animals had lower CD31-labeled vasculature/area (Fig. 3B) in both Group 4wk ((5.3%) versus controls (7.4%), p = 0.044) and Group 4wk + p ((4.5%) vs controls (7.4%), p = 0.007) indicating a reduction in angiogenesis in the placenta of animals treated long-term with imatinib, even when the drug was stopped prior to pregnancy (4wk).

## Discussion

This study was designed as an initial investigation into the effect of long-term imatinib on placentation and embryo development. As a first step, we have examined epigenetic and histologic profiles of the developing placenta in a mouse model. This model represents cancer survivors who’ve been treated long-term with adjuvant imatinib therapy prior to pregnancy. Imatinib is a tyrosine kinase inhibitor, a rapidly expanding class of chemotherapeutic drugs used commonly in cancers affecting reproductive aged women. Previous studies have called into question the potential negative impact on fertility potential and pregnancy but the data regarding the effect of TKI’s before and during early embryogenesis are inconclusive^14,25,26^. This study demonstrated epigenetic dysregulation of key placental genes among mice receiving imatinib both before and during pregnancy. These results demonstrate a potential negative impact even when imatinib is stopped prior to pregnancy.

In our study, we treated mice long-term, then mated females immediately after stopping imatinib, and found a significant detrimental effect on placental development. Vascular development in the placenta was significantly reduced, while labyrinth size and trophoblast layer width also tended to be smaller when females were exposed to imatinib prior to pregnancy. The question arises, if there were a prolonged wash-out period, a time without imatinib prior to pregnancy, would this prevent the effects on placenta. Unfortunately, patients who have stopped taking imatinib prior to pregnancy, have a high likelihood of cancer returning (>60%)^18^. For women whose cancer progresses during pregnancy, they are left with a very distressing question on whether to resume imatinib or wait until after the baby is born, a choice that risks both the child and the mother. Because of this difficult choice between taking a teratogenic drug versus cancer progression, there are no official medical recommendations regarding a potential wash-out period for cancer survivors taking imatinib and seeking pregnancy. Our research is aimed at helping to provide information on the potential effects of imatinib prior to pregnancy. By understanding the possible effects, future studies may be able to find treatments that would offer protections for cancer survivors during pregnancy.

The primary targets for imatinib are the ABL-family of tyrosine kinases, although it is not known if these kinases play a role in placentation. We hypothesized that the collateral affinity of imatinib to inhibit tyrosine kinases such as KIT and PDGFR may have led to the observed placental aberrations. KIT and PDGRF are both critical for normal fetal development, angiogenesis, and placentation^27–29^.

The histological analysis of the placental specimens demonstrated a clear relationship between imatinib treatments and a decreased placental size. More specifically, it was observed that the labyrinth area of the placenta was particularly affected by imatinib exposure. It was further discovered that imatinib treatments resulted in diminished angiogenesis in the placental labyrinth, even when imatinib was stopped prior to pregnancy indicating a possible effect of long-term imatinib treatments on the uterine environment. Inhibition of angiogenesis is one of the functions of imatinib to stop cancerous growth and it appears that there is potential for a similar effect in the developing placenta^7^. Poor vascularization of the labyrinth may be one mechanism accounting for reduced placental growth and decrease in fetal numbers at E13. Average fetal weights were not different from controls, but our measurements were taken at an early stage of pregnancy (E13). Poor placental development may have a more significant effect on fetal growth later in pregnancy and should be further examined.

In this study, we investigated specific genes that have known associations to phenotypic abnormalities when inappropriately regulated in the placenta. Specifically, we examined the differential methylation of the imprinted genes *H19*, *Peg1*, *Peg3*, *Snrpn*, *Cdkn1c* (*Kv or Lit1)*, and *Igf2*. Interestingly, *Ig DMR*, *Peg1 DMR*, *Peg3 DMR*, and *Snrpn DMR* were hypomethylated in the 4wk group which received imatinib prior to pregnancy. Given the significant effects of pre-pregnancy imatinib, it was surprising that methylation of these genes in the 4wk + p group who were exposed to imatinib before and during pregnancy were not significant. However, mice in the 4wk + p group also had significantly fewer implantations at E13, indicating a loss of embryos very early in pregnancy, possibly before implantation or even a reduction in follicle development, ovulation or fertilization. We theorize that embryos with significant methylation aberrations were more susceptible to the continuous imatinib exposure during pregnancy and that these embryos are the ones lost early, thus the non-significant findings of methylation errors at E13. Additional studies into the mechanisms of imatinib action on embryo and placental development will be needed to further determine the cause of this outcome.

Similar DNA hypomethylation errors of *Cdkn1c*, *Igf2*/*H19*, and *Snrpn* genes are associated with developmental diseases such as Beckwith-Wiedemann (BWD) and Angelman Syndromes^30,31^. Placental aberrations have also been reported in association with these epigenetic changes in BWD. Hypomethylation of *Cdkn1c* (*p57*^*KIP2*^) is associated with excessive growth of the extravillous trophoblast in human placenta. The similarity of hypomethylation detected in our study of mouse placenta to those that are known to cause serious developmental diseases in humans emphasizes the importance for future research to examine possible effects of pre-pregnancy imatinib on the development of offspring.

*Peg1* and *Peg3* are important for maternal care behavior and hypomethylation has demonstrated a deficiency in actions such as milk-letdown, nest building and placentophagia^32,33^. Peg3 in particular is a marker of vascular endothelial stem cells during fetal development^34^. A reduction in Peg3 would correlate with the reduction in neo-vascular growth, although we did not actually detect a reduction at the RNA level. Meanwhile, *Snrpn* encodes a small nuclear ribonucleoprotein complex essential for pre-mRNA processing. Altered epigenetic regulation can result in growth defects or lethality. How imatinib is affecting DNA methylation is unknown, but demethylation of key genes by imatinib has been reported previously^35^. A recent study found that knockdown of tyrosine kinases by nilotinib, a drug closely related to imatinib caused reduced expression of DNA methyltransferases leading to global DNA hypomethylation^36^. In our study we used the LUMA assay to measure global DNA methylation but were unable to detect a significant global change at E13. Future studies will need to examine gene expression at the protein level in addition to RNA, and additional time points during development are needed. It should be noted that there is evidence that the placenta may be more sensitive than the fetus to an early insult of epigenetic dysregulation^21,37^. Therefore, it cannot be assumed that epigenetic dysregulation in the placenta necessarily equates to a disturbance of the epigenome of the fetus. More studies are needed to better determine the full effects of tyrosine kinase inhibitors on pregnancy and development.

This study’s clinical impact is strengthened by the high conservation of DNA methylation patterns between the human and mouse developing embryo^38^. A possible biological link was detected between the epigenetic results and the histological analysis of the placentas. The careful dose titration in our animal model represents a dose range equivalent to that in humans undergoing chemotherapy. One limitation that may confound placental studies, is the effect of maternal derived decidual contamination^28^. However, meticulous dissection of the placental bed minimized the extent of this potential confounder. An additional limitation is that females in the 4wk + p group were treated with imatinib prior to, and during ovulation in addition to pregnancy. So the effects of imatinib may have also impacted follicle, oocyte or preimplantation development. Larger studies are needed to determine the specific targets of imatinib prior to and during pregnancy and whether the long-term imatinib affected oocyte quality. It will be important to know if cryopreservation of gametes prior to imatinib treatments (ie, Fertility Preservation Treatments) would prevent the placental aberrations that were identified in the present model.

It is notable that women undergoing Assisted Reproductive Technology (ART) also experience aberrant methylation at similar ICRs as those demonstrated in this study^22,24^. This is of particular importance to women undergoing cancer treatment as they often need ART for their therapy induced infertility^39^. Should these same women also be treated with imatinib, they could potentially experience a compounded effect. Certain ICRs such as *H19*, *Igf2*, *Peg3* and *Cdkn1c/Kcnq1ot1 (KvDMR*) are particularly sensitive to *in vitro* conditions and other environmental (external) factors^22^.

## Summary

Histological analysis of placentas demonstrated minor but significant growth abnormalities leading to a reduction in the size of the placenta; more specifically the size of the labyrinth layer and reduced angiogenesis. Changes were seen even when imatinib was stopped prior to pregnancy and were exacerbated when imatinib was continued during pregnancy. Problems with placental development and implantation may be due to long-term effects of imatinib on the uterus or ovary. Additional studies are needed to assess whether imatinib affected oocyte quality which could lead to developmental effects in the embryo and could be the cause of fewer implantations seen at E13. Taken together, the epigenetic data and histological data suggest a long-term negative impact on placentation in mice exposed to imatinib. An important question that still needs to be examined is whether placental development would return to normal, if there were an extended time period (wash-out) between stopping the drug treatment and the beginning of pregnancy. Unfortunately for patients, a prolonged cessation of treatment can increase the likelihood of cancer recurrence so these questions are important concerns for our patients.

This pilot study detected dysregulation of epigenetic methylation of critical placental genes that may impact development of the fetus. This study also supports one plausible underlying mechanism which would link growth aberrations to diminished angiogenesis in the labyrinth zone of the placenta. This study demonstrated a potential for prolonged effects of imatinib during pregnancy, but further research is needed to determine if the effects seen in this animal model, also occur in patients. Women who’ve been treated with imatinib may be at higher risk of placental insufficiency with the accompanying concerns for fetal development and possibility of preterm birth.

## Methods

### Mouse model

Mice were purchased from Envigo, USA (formerly Harlan), housed in a temperature and light cycle controlled animal facility. All experiments were conducted in accordance with the”Guide for the Care and Use of Laboratory Animals” and preapproved by the University of Southern California Institutional Animal Care and Use Committee^40^. Young adult female mice (CF1 strain), aged 7 weeks old were assigned to each of three treatment groups (Table 1). Treatment Group 1 (4wk): daily dose of 400 mg/kg of intraperitoneal (100 µl, IP) imatinib plus imatinib ad lib in drinking water (1 mg/ml) for four weeks. Group 2 (4wk + p): The same treatment as in Group 1, plus imatinib was continued ad lib in their drinking water throughout pregnancy. Controls: injected IP with 100 µl of sterile water (diluent) daily for four weeks. Imatinib was purchased from Tocris Biotechne and prepared daily, by mixing in sterile water followed by 0.22um filtration. Water bottles were refreshed daily and completely changed for fresh once each week. There were total of 30 female mice assigned to this study. Unfortunately, one control female died from unrelated causes when a valve in the watering system failed to function. Therefore, there were n = 8 (controls), n = 9 (4wk), and n = 12 (4wk + p) female mice in this study.

Following the 4 weeks of ip injections, females were caged with adult B6D2F1 males of proven fertility; 1–2 females to each male. Females were checked each morning for the presence of a vaginal plug that indicated mating. Individual females were housed alone throughout pregnancy and provided with paper houses and nesting materials. Males were not exposed to imatinib. At E13, females were euthanized by fluorothane inhalation and cervical dislocation. Maternal blood was drawn via cardiac puncture at the time of euthanasia and their uteri were harvested. Uteri were either fixed intact in 10% formalin or dissected. The dissected uteri were examined fresh, under a dissecting microscope, the number of implantations, resorptions, and gross morphology of the embryos were recorded. Three to six placentas from each female were dissected, flash-frozen on dry ice, and stored at −80C for later molecular testing. Blood samples were centrifuged at 1,000 g for 10 minutes. Serum was collected from the top of the tubes, transferred to microcentrifuge tubes, and stored at −80C until thawed for hormone assays.

### LC-MS/MS measurement of imatinib and N-desmethyl imatinib in serum

To determine the serum levels of imatinib and its primary metabolite N-desmethyl imatinib (CGP71588) following treatments either by IP injection or ad lib in drinking water LC/MS-MS analysis was performed by the Bioanalytical Shared Resource/Pharmacokinetics Core Facility at the Oregon Health and Science University, using a slight modification of the protocol previously published^41^. Briefly, serum samples were thawed and extracted with acetonitrile containing internal standards (d_8_-imatinib and d_8_-desmethyl imatinib). Following centrifugation to remove protein and filtration the supernatants were analyzed using an SCIEX 5500 QTRAP triple–quadrupole hybrid linear ion trap mass spectrometer interfaced to a Shimadzu Prominence HPLC system. The instrument was operated in positive ESI mode and compounds were detected and quantified with multiple reaction monitoring (MRM) using published transitions. The limit of quantification was 1 ng/ml for imatinib and desmethyl imatinib. (For more details on the LC-MS/MS study, see Supplementary Fig. S1). This study used five females in each group plus, one negative (untreated) control.

#### **Hormone assays**

Estrogen and progesterone were measured in maternal serum using Immulite 2000 immunoassay analyzer from Siemens Healthcare Diagnostics. Estradiol 2000 and progesterone 2000 immunoassay kits were run according to manufacturer protocols. The two assays required a minimum of 300 μl of serum therefore only those samples with >300 μl could be assayed.

### DNA Isolation from placental tissue

DNA was extracted from snap-frozen placentas utilizing phenol-chloroform and TRIzol (Invitrogen). Each placenta was homogenized in lysis buffer (50 mM Tris, pH 8.0, 100 mM EDTA, 0.5% SDS + proteinase K). The samples were incubated for 90 minutes followed by phenol-chloroform extraction for genomic DNA isolation.

### Epigenetic analysis- bisulfite pyrosequencing and luminometric methylation assay

Epigenetic analysis was carried out on placental DNA extracted from E13 females using methods previously described^24,42^. DNA methylation imprinting control regions previously found to be adversely affected by environmental conditions such as culture and embryo transfer^21,24,43^. These included *H19 DMR* (6 CpG), *Ig DMR* (5 CpG), *Peg1 DMR* (5 CpG), *Peg3 DMR* (6 CpG), *KvDMR* (6 CpG), *Snrpn DMR* (7 CpG), *Igf2 DMR1* (4 CpG) were measured by using bisulfite pyrosequencing (Epitect: Qiagen). A luminometric methylation assay (LUMA) analyzed global methylation profiles as previously described^44^. This was done in order to observe if the epigenetic defect was a result of globally impaired DNA methylation machinery or a locus specific effect.

### Histological analysis

Histological analysis was undertaken to evaluate imatinib’s effect on implantation and placental development. Uterine and placental tissues were aseptically collected and fixed in 10% buffered formalin for at least 24 h for histological assessment. The samples were cut in halves midsagittal and processed in Tissue Tek VIP tissue processor, embedded in paraffin wax and serially sectioned at 5 μm thickness for 100 slides for each placenta. Every 10^th^ slide was processed by standard hematoxylin and eosin histological methods. To visualize the histological samples, the slides were loaded into a Hammamatsu Nanozoomer 2.0 HT and scanned with NDP.2.5 software. Images of samples were assessed utilizing NDP.view.2 software, which allowed for all the tissue layers to be measured accurately. Quantitation of the morphology was carried out by taking measurements of 6–8 placenta of each treatment group. Measurements including the size, width and perimeter of the whole placenta, the labyrinth zone, the junctional zone and the decidual zone were taken and compared between treatment groups (n = 6–8 placentas per group, N = 4 animals per group).

### Immunohistochemistry and quantifications

Immunohistochemistry was performed to measure vascularization of the labyrinth zone. We used the angiogenic marker CD31 (Abcam AB28364) according to a previously established protocol^45^. Briefly, sections were deparaffinized and rehydrated in 3 xylene solutions, then 100%, 90%, 80% and 70% ethanol, and water for 5 minutes each. Antigen retrieval was performed by heating tissue sections in 1X Dako solution (Dako Denmark) for 20 minutes. Endogenous peroxidase activity was blocked in methanol containing 3% hydrogen peroxide for 15 minutes. After blocking with 5% normal goat serum (Vector Laboratories) for 60 minutes at room temperature, sections were incubated with a rabbit anti-CD31 antibody (1:100) at 4 °C overnight. After washing with PBST, sections were incubated with biotinylated anti-rabbit secondary antibody (Jackson Immuno Research, 1:200) for 60 minutes at room temperature. After incubation with ABC solution (Vector Laboratories) for 30 minutes, sections were reacted with 3, 3′-diaminobenzidine (DAB) Peroxidase Substrate (Vector Laboratories) for 30 seconds and counterstained with hematoxylin. CD31 positive vasculature was quantified using FIJI software^46^. Slides were scanned and visualized using Nanozoomer. For each placenta at 40X magnification, 5 random areas of each layer were selected and analyzed using the Trainable Weka Segmentation plugin. Each image was separated into 3 classes: Class 1 white for “white background”, Class 2 blue or light blue area for “unstained area” and Class 3 brown area for “positively stained area”. Once the program was trained, a probability map was generated for each image (5 per layer per section) and a threshold was set to match with the original Nanozoomer image. A measurement of Class 3 “positively stained area” was taken for quantitation of the percentage of positively stained area over the total size of the image. 6–8 placentas of each treatment group was analyzed.

### Gene expression analysis

To isolate total RNA, we added 500 µl of Trizol Reagent (Abion and Sigma Life Sciences) to 15 mg of frozen placenta and homogenized for 30 seconds using 1.5 mm molecular grade Zirconium beads and a BeadBug microtutube homogenizer (Benchmark Scientific). The liquid sample was transferred to a clean tube and total RNA isolated by standard chloroform protocol^47^. RNA integrity and quantity was assessed with a picochip on an Agilent Bioanalyzer and real time PCR conducted on a Studio Quant 7 Real Time PCR System (Applied Biosystems) by staff in the USC Norris Genomics Core Laboratory. Validated PCR primer-probe sets were purchased from Applied Biosystems (Supplementary Table S2). Samples were measured in triplicate and delta Ct values calculated and statistically analyzed as reported previously^48^.

### Statistical analysis

One-way analysis of variance (ANOVA) was used to analyze pregnancy data. Epigenetic data were non parametric and was thus evaluated using a Kruskal-Wallis test. Pairwise comparison between the study groups of interest and the control group was done using a Wilcoxon rank sum test. For histological data, multiple comparisons conducted between normally distributed experimental groups were analyzed by one-way ANOVA followed by Dunnett’s test comparison and standard error of the means (SEM). Multiple comparison between non-normally distributed groups was made using Kruskal Wallis tests when appropriate. The statistical significance was assigned at *p* ≤ 0.05. All analyses were done using STATA version 12.1 (College Station, Tx) or GraphPad Prism software 7 (GraphPad Prism, San Diego, CA).

## Supplementary information

Supplementary Data
