# Supplementary material for: Imatinib treatments have long-term impact on placentation and embryo survival
Source: Sci Rep. 2019 Feb 22;9:2535. doi: 10.1038/s41598-019-39134-0 (PMC6385245; doi:10.1038/s41598-019-39134-0)
Supplement: Supplementary file 1 — Supplementary Data [file 41598_2019_39134_MOESM1_ESM.docx]

**Imatinib has long-term impact on placentation and embryo survival**

Wael Salem^1^, Kailiang Li^3^, Christopher Krapp^4^, Sue Ann Ingles^2^, Marisa S Bartolomei^4^, Karine Chung^1^, Richard J Paulson^1^, Romana A Nowak^3^, Lynda K McGinnis^1,5^

*****************

Department of Obstetrics and Gynecology^1^, Division of Reproductive Endocrinology and Infertility^1^, Department of Preventative Medicine^2^, University of Southern California, Los Angeles CA USA

Department of Animal Sciences^3^, University of Illinois, Urbana-Champaign IL USA

Department of Cell and Developmental Biology^4^, University of Pennsylvania, Philadelphia PA USA

**************************************************************************************

**Supplemental Figure S1: LC-MS/MS analysis of imatinib levels in serum**


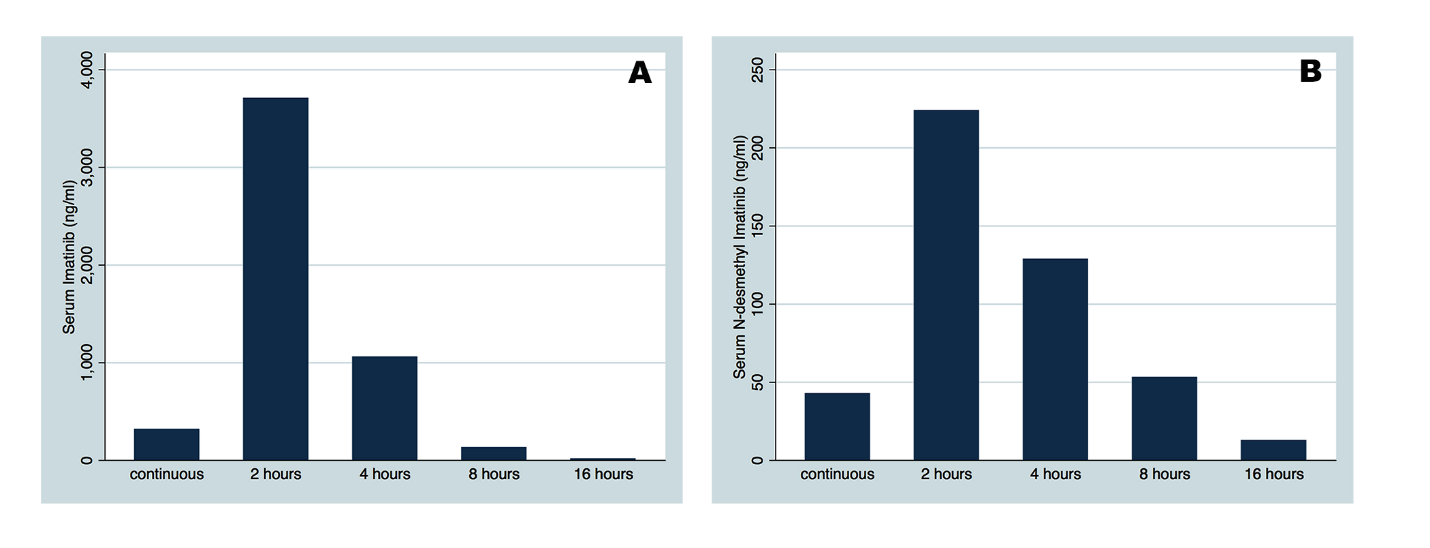


***Serum levels of imatinib***

Blood levels of imatinib are known to decline relatively rapidly in the mouse model when animals are treated by daily IP injections. One previous study had reported the use of imatinib *ad lib* in drinking water, with a significant effect on tumor regression. They counted the total number of primordial, primary, and secondary follicles in ovaries after 2 months of treatment. While they detected no significant differences in the total numbers follicles, they did report that imatinib treated ovaries were disorganized, lacking the usual order of primordials at the cortex with increasing follicle sizes associated with deeper location within the ovary^1^. Unfortunately, they did not provide extensive details of the ovarian histology. Since their treatment lead to reduction in tumor size, it was assumed that this route of administration would maintain imatinib at high levels in the blood. However, they did not actually measure the levels of imatinib attained. Therefore, we began our studies by using liquid chromatography tandem mass spectrometry (LC-MS/MS) analysis to measure the levels of imatinib and its primary metabolite (N-desmethyl imatinib) in serum. Mice were treated with 1mg/ml *ad lib* in the drinking water (**continuous**) or injected IP with a single dose 400mg/kg (Supplementary Fig. S1 online). Blood was collected on day 7 of continuous imatinib ad lib in drinking water, or 2, 4, 8, or 16 hours after IP injections. Five females in each group plus one control mouse that received no imatinib, therefore a total of 26 mice were used.

**Results**

Serum levels of imatinib were high at 2h post-injection, being above the clinical therapeutic level of 1,000 ng/ml. However, serum levels declined rapidly, becoming nearly undetectable by 16h. Imatinib *ad lib* in the drinking water produced serum levels approximately equivalent to 6-8h post-injection. N-desmethyl imatinib is the primary active metabolite of imatinib^2^. Although little is known about the specific actions of this metabolite, it does interact with targets of imatinib and is metabolically active^3^, therefore we also measured the levels of N-desmethyl imatinib. This metabolite also declined rapidly in serum after IP injections, but less rapidly than imatinib itself and N-desmethyl imatinib was still measurable at 16h. Exposure through drinking water *ad lib* produced serum levels equivalent to approximately 6-8h post-injection. Based on these preliminary data, mice in this study were treated with a combination of daily IP injection of imatinib to raise the serum levels above 1,000ng/ml (therapeutic levels for patients), plus imatinib ad lib to maintain imatinib at measurable levels long-term.

**Fig. S1. References**

1 Schultheis, B., Nijmeijer, B. A., Yin, H., Gosden, R. G. & Melo, J. V. Imatinib mesylate at therapeutic doses has no impact on folliculogenesis or spermatogenesis in a leukaemic mouse model. *Leukemia research* **36**, 271-274, doi:10.1016/j.leukres.2011.09.025 (2012).

2 Gschwind, H. P. *et al.* Metabolism and disposition of imatinib mesylate in healthy volunteers. *Drug Metab Dispos* **33**, 1503-1512, doi:10.1124/dmd.105.004283 (2005).

3 Mlejnek, P., Dolezel, P., Faber, E. & Kosztyu, P. Interactions of N-desmethyl imatinib, an active metabolite of imatinib, with P-glycoprotein in human leukemia cells. *Annals of hematology* **90**, 837-842, doi:10.1007/s00277-010-1142-7 (2011).

**Supplementary Table S1. RNA levels from differentially methylated genes**

**were not significantly affected in E13 placenta**

To determine if the changes in DNA methylation resulted in detectable changes in gene expression in the E13 placental tissues, we used Real Time qPCR analysis to determine the levels of RNA (see methods section). The *H19 DMR, Igf2 DMR, Peg3 DMR,* and *Kv DMR* control the expression (RNA) of *H19*, *Igf2, Peg3, Cdkn1c,* and the long non-coding RNA *Kcnq1ot1.* Therefore, these genes were selected for analysis. No significant differences were detected in the levels of RNA for these genes at E13.

|  | **Controls** | | **4wk** | | | **4wk+p** | | |  |
| --- | --- | --- | --- | --- | --- | --- | --- | --- | --- |
|  | Delta^2^ Ct | | Delta^2^ Ct | | Fold-change | Delta^2^ Ct | | Fold -change |  |
| Genes | mean | SD | mean | SD | (vs. control) | mean | SD | (vs. control) | p value |
|  |  |  |  |  |  |  |  |  |  |
| CDKN1C | 3.27 | 0.86 | 2.85 | 0.88 | 1.33 | 3.07 | 0.88 | 1.15 | 0.72 |
| kcnq1ot1 | 2.50 | 0.85 | 2.89 | 1.29 | 0.76 | 3.12 | 0.67 | 0.65 | 0.54 |
| IGF2 | -0.05 | 0.60 | 0.21 | 0.66 | 0.84 | 0.18 | 0.23 | 0.85 | 0.66 |
| H19 | -3.07 | 0.38 | -2.68 | 0.63 | 0.76 | -2.66 | 0.34 | 0.75 | 0.26 |
| PEG3 | -1.05 | 1.08 | -1.21 | 1.26 | 1.12 | -0.40 | 1.37 | 0.64 | 0.51 |

Values are show as means +/- SD; Mean delta Ct values were compared using ANOVA

**Supplementary Table S2. Real Time-PCR primers**

| **Gene** | **Assay ID** | **Amplicon Length** | **Exon Boundary (probe)** |
| --- | --- | --- | --- |
| *Cdkn1c* | Mm00438170_m1 | 115 | 2-3 |
| *H19* | Mm01156721_g1 | 78 | 3-4 |
| *Igf2* | Mm00439564_m1 | 107 | 2-3 |
| *Kcnq1ot1* | Mm03935655_s1 | 88 | 1-1 |
| *Peg3* | Mm01337379_m1 | 60 | 7-8 |
| *Gapdh* (control) | Mm99999915_g1 | 107 | 2-3 |

Validated primer-probe sets were purchased from Applied Biosystems
